# Prognostic Factors for 28-Day Mortality in Pediatric Patients with Acute Leukemia and Candidemia Following Intensive Chemotherapy: A Retrospective Study

**DOI:** 10.3390/hematolrep17040038

**Published:** 2025-07-30

**Authors:** Tran Thi Kieu My, Hoang Thi Hong, Mai Lan, Tran Quynh Mai, Dang Hoang Hai, Ta Thi Dieu Ngan

**Affiliations:** 1 Department of Hematology, Hanoi Medical University, Hanoi 10000, Vietnam; hoangthihong@hmu.edu.vn (H.T.H.); tadieungan@hmu.edu.vn (T.T.D.N.); 2 National Institute of Hematology and Blood Transfusion, Hanoi 10000, Vietnam; mai_lan_1009@yahoo.com (M.L.); tranquynh.mai996@gmail.com (T.Q.M.); danghoanghai1294@gmail.com (D.H.H.)

**Keywords:** candidemia, leukemia, pediatric, 28-day mortality, granulocyte transfusion, antifungal prophylaxis, absolute lymphocyte count

## Abstract

**Background/Objective:** Candidemia is a serious complication following intensive chemotherapy and is associated with high mortality in pediatric patients. This study aimed to identify the factors associated with 28-day mortality in pediatric patients with candidemia. **Methods:** We retrospectively analyzed 63 pediatric patients diagnosed with acute leukemia and candidemia following intensive chemotherapy. Clinical characteristics, laboratory findings, and epidemiological data were collected. Antifungal susceptibility data were available for 60 patients. Kaplan–Meier survival analysis was used to estimate the 28-day mortality rate, and Cox regression was performed to identify prognostic factors. **Results:** The 28-day mortality rate among the 63 patients (57.1% male, median age 9.74 years) was 36.5%. *Candida tropicalis* was the predominant species (96.8%). Antifungal susceptibility rates were 100% for amphotericin B and caspofungin and 22.2% for fluconazole. The factors independently associated with reduced 28-day mortality were an absolute lymphocyte count (ALC) ≥ 0.2 G/L at the time of candidemia diagnosis (5.3% vs. 50% mortality; hazard ratio [HR] = 0.08; 95% confidence interval [CI], 0.01–0.61), the use of antifungal prophylaxis (AFP) (26.3% vs. 52%; HR 0.31; 95% CI, 0.13–0.74), and granulocyte transfusion (GTX) combined with granulocyte colony-stimulating factor (G-CSF) (20% vs. 47.4%; HR = 0.31; 95% CI, 0.11–0.85). **Conclusions:** Our findings suggest that an ALC ≥ 0.2 G/L, AFP, and the administration of a GTX combined with G-CSF may be considered favorable prognostic factors.

## 1. Introduction

Over the past two decades, advancements in intensive chemotherapy protocols, supportive care, and novel therapies have significantly improved the survival rates of pediatric patients with acute leukemia [1,2]. However, treatment-related mortality (TRM) continues to pose a considerable challenge, with rates as high as 9.21% in lower-middle-income countries and even higher rates, reaching 14.19%, in low-income countries, where infections represent the leading cause of death [3]. Numerous studies have examined TRM in pediatric patients with acute leukemia, highlighting the critical role of supportive care in enhancing overall survival [4].

The incidence rate of invasive fungal infection (IFI) among pediatric patients with cancer ranges from 2 to 21% [5]. Invasive candidiasis remains one of the most common causes of IFI in this population and is associated with a poor prognosis, with reported mortality rates between 20% and 70% [6]. The diagnosis and management of IFI should be informed by local epidemiology, available resources, and institutional clinical practices [7]. Notably, the epidemiology of *Candida* spp. can vary across regions, countries, and even among healthcare facilities within the same country.

The use of AFP is crucial for preventing candidemia and improving survival outcomes in high-risk populations. AFP with azoles (such as fluconazole or posaconazole), echinocandins, or amphotericin B is recommended for pediatric patients at high risk of IFI (incidence > 10%), including patients diagnosed with acute myeloid leukemia (AML), relapsed/refractory (R/R) acute leukemia, or acute lymphoblastic leukemia (ALL) during clinical phases involving prolonged glucocorticosteroid therapy or persistent profound granulocytopenia [7]. Current research suggests that the development of lymphocyte responses plays a crucial role in mediating host defense against *Candida* infections [8]. Drummond et al. (2022) highlighted the role of T-helper 17 (Th17) cells in reducing the initial fungal burden in invasive candidiasis following the disruption of the gastrointestinal mucosal barrier [9]. Conversely, Pappas et al. (2018) emphasized that despite the central role Th17 cells play in mucosal candidiasis, effective immune responses in invasive candidiasis primarily rely on neutrophils and mononuclear phagocytes [10].

Given the profound depletion of myeloid cells that often occurs following intensive chemotherapy in patients with acute leukemia, this study was conducted to evaluate the prognostic significance of ALCs for mortality in this specific clinical context. Since the 1970s, GTXs have been used to treat neutropenic patients with refractory infections. Several studies, including those by Netelenbos et al. (2019) and West et al. (2017), have suggested that a GTX may be considered in patients with absolute neutrophil count (ANC) levels of <0.5 G/L persisting for at least 72 h, accompanied by a life-threatening infection that is unresponsive to systemic antimicrobial therapy for at least 48 h [11,12]. The combination of GTX and G-CSF has been used as adjunctive therapy in IFI management in pediatric patients [12]. In our institution, this strategy has been applied as a salvage therapy for life-threatening infectious complications associated with severe neutropenia. Therefore, in this study, we aimed to evaluate the factors influencing the prognosis of pediatric patients with candidemia.

## 2. Materials and Methods

### 2.1. Study Design

**Figure 1:**
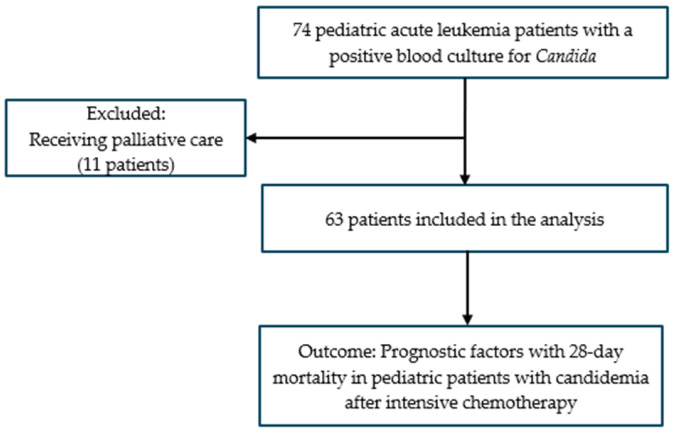
Flowchart for the enrolled patients.

This retrospective, single-center observational study was conducted at the Department of Pediatric Hematology, National Institute of Hematology and Blood Transfusion, between January 2022 and December 2024. Eligible patients were those under 16 years of age diagnosed with either non-M3 AML or ALL who had received intensive chemotherapy. Patients with secondary AML or ALL, acute leukemia of ambiguous lineage, prior allogeneic hematopoietic stem cell transplantation, underlying congenital diseases, genetic disorders, or malformations were excluded from the study. Among the 74 pediatric patients enrolled in this study and diagnosed with a first episode of candidemia, 11 patients receiving palliative care were excluded from the analysis as our objective was to evaluate prognostic factors in pediatric acute leukemia patients with candidemia following intensive chemotherapy (Figure 1). Antifungal susceptibility data were available for 60 out of the 63 patients, as 3 cases had *Candida*-positive blood cultures identified only after death.

### 2.2. Candidemia Diagnosis and Data Collection

For patients with febrile neutropenia, 2–10 mL of peripheral venous blood was collected for bacterial and fungal cultures. Pathogen identification and antifungal susceptibility testing were performed using the VITEK 2 Compact system. ANC and ALC values were recorded from blood samples collected within 24 h before or after the time of candidemia diagnosis. Clinical characteristics were recorded at the time of candidemia diagnosis.

### 2.3. Definitions

Proven invasive fungal infection was defined according to the criteria established by the European Organization for Research and Treatment of Cancer/Invasive Fungal Infections Cooperative Group and the National Institute of Allergy and Infectious Diseases Mycoses Study Group 2008 [13]. The time of candidemia diagnosis was defined as the time of blood culture collection from which *Candida* spp. was isolated. Febrile neutropenia was defined as a single oral temperature of >38.3 °C or a sustained temperature of >38.0 °C for more than 1 h, with an ANC value of <0.5 G/L or one expected to decrease to <0.5 G/L [14]. Neutropenia was classified as severe (<0.5 G/L) or profound (<0.1 G/L) [15]. Grade 4 lymphopenia was defined as an ALC value of <0.2 G/L. Body mass index (BMI) categories were defined according to World Health Organization criteria [16,17]. The remission criteria for AML and ALL were defined according to the recommendations of the European LeukemiaNet [18,19]. GTX were considered for patients with persistent neutropenia (ANC < 0.5 G/L for ≥72 h) and life-threatening infections that were unresponsive to systemic antimicrobial therapy for ≥48 h, with their units derived from pooled whole-blood donations. GTX initiated within 7 days of candidemia diagnosis were considered eligible for analysis.

### 2.4. Statistical Analysis

Categorical variables are reported as counts and percentages, while continuous variables with non-normal distributions are summarized using the median and range (minimum–maximum). The 28-day mortality following candidemia was estimated using Kaplan–Meier survival curves, with *p* values calculated based on the log-rank test. Cox regression was employed to analyze the factors associated with 28-day mortality, and hazard ratios with 95% confidence intervals were determined. The multivariate Cox regression model included the factors independently associated with 28-day mortality as identified in univariate analysis. A two-sided *p*-value of less than 0.05 was deemed statistically significant. Statistical analysis was performed using IBM SPSS Statistics, version 26.0 (IBM Corp., Armonk, NY, USA).

## 3. Results

In total, 63 patients with acute leukemia who were diagnosed with candidemia following intensive chemotherapy were included in this analysis (Figure 1). The baseline characteristics of the study population are summarized in Table 1. At the time of candidemia diagnosis, 61 patients (96.8%) had profound neutropenia, and 44 patients (69.8%) exhibited Grade 4 lymphopenia. Epidemiological and treatment-related characteristics are presented in Table 2.

**Figure 2:**
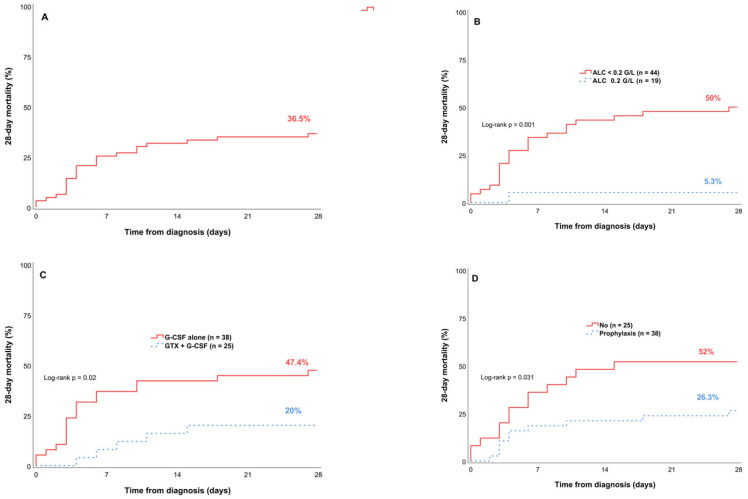
Kaplan–Meier survival curves for 28-day mortality: (**A**) entire cohort; (**B**) stratified by ALC; (**C**) stratified by GTX; (**D**) stratified by AFP.

Table 3 presents the analysis results of the factors associated with the 28-day mortality rates, which are visualized in Figure 2 using Kaplan–Meier survival curves. Cox regression was performed for univariate and multivariate analyses of the associated factors. Multivariate Cox regression revealed three factors independently associated with 28-day mortality: ALC at the time of candidemia diagnosis (ALC ≥ 0.2 G/L vs. ALC < 0.2 G/L: 5.3% vs. 50%; HR = 0.08; 95% CI, 0.01–0.61; *p* = 0.015); AFP use (yes vs. no: 26.3% vs. 52%; HR 0.31; 95% CI, 0.13–0.74, *p* = 0.008); and the combination of a GTX and G-CSF (GTX + G-CSF vs. G-CSF alone: 20% vs. 47.4%; HR = 0.31; 95% CI, 0.11–0.85), *p* = 0.024).

## 4. Discussion

This retrospective observational study involved 63 pediatric patients with acute leukemia and candidemia following intensive chemotherapy. The predominant species identified was *C. tropicalis*, which exhibited a low susceptibility rate to fluconazole. In a 2017 review by Pana et al. on pediatric IFI in the United States, *Candida albicans* was the most frequently isolated species, accounting for 38.1% to 55.3% of cases. However, in pediatric cancer populations, a higher proportion of non-albicans *Candida* species, such as *C. tropicalis* and *Candida parapsilosis*, has been identified [20].

The high prevalence of *C. tropicalis* (96.8%) in our study may be influenced by underlying host conditions and regional epidemiological patterns. In the review article of the epidemiology of IFI in Asian patients with hematological malignancies by Iyadorai et al. (2024), *C. tropicalis* accounted for 46.2% of IFI cases in tropical countries and emerged as the predominant pathogen in Malaysia—a Southeast Asian country similar to Vietnam—after 2010 [21]. In addition, the use of central venous catheters (CVCs) is a recognized risk factor for *C. albicans* bloodstream infections in cancer patients [6]. However, as CVCs were rarely used in our department, this may have contributed to the predominance of *C. tropicalis*. According to Whaley et al. (2017), *C. tropicalis* accounted for 20% to 45% of *Candida* spp. isolates in the Asia–Pacific region and was most frequently observed in patients with hematologic malignancies, particularly AML [22]. In contrast, in North America, Europe, and the Middle East, the predominant species isolated were *C. albicans* and *Candida glabrata* [22]. A multicenter study by Tan et al. (2016) involving 861 patients in the Asia–Pacific region found that *C. tropicalis* was the second most prevalent *Candida* species isolated (30.7%) [23]. Susceptibility to caspofungin was as high as 99.6%, whereas susceptibility to fluconazole was markedly lower, ranging from 61.7% to 85.7% [23]. The increased use of fluconazole prophylaxis over the past two decades may partly explain the emergence of fluconazole-resistant *Candida* isolates [24].

The 28-day mortality rate among patients with candidemia in our cohort was 36.5%. All patients were severely immunocompromised due to underlying acute leukemia and chemotherapy-related toxicity, with 61 patients (95.3%) having an ANC level of <0.1 G/L at the time of candidemia diagnosis. The reported mortality rates following candidemia in patients with hematologic or solid malignancies have exceeded 30% in multiple studies [5,25,26]. *C. tropicalis* is a highly virulent pathogen and has been associated with poor clinical outcomes. In the multicenter European study ECMM Candida III (2023), which involved 632 patients with candidemia, the 90-day mortality rate reached 42.9%. Multivariate analysis identified bloodstream infection with *C. tropicalis* (*n* = 44) as an independent baseline predictor of mortality [27].

In our study, univariate and multivariate analyses of baseline characteristics, laboratory findings, epidemiological data, and treatment-related variables revealed only three factors significantly associated with 28-day mortality: AFP, the combination of GTX with G-CSF, and the use of ALC at the time of candidemia diagnosis.

AFP analysis showed a significantly lower 28-day mortality rate among patients who received prophylaxis than those who did not, with rates of 26.3% versus 52% (HR 0.31; 95% CI, 0.13–0.74; *p* = 0.008). The use of AFP in pediatric patients with cancer is strongly recommended in the ECIL-8 guidelines [7]. Marr et al. (2000) reported that fluconazole prophylaxis in allogeneic hematopoietic stem cell transplant recipients significantly reduced the incidence of invasive candidiasis and the associated mortality among those who developed the infection [28].

Neutropenia is a well-established significant risk factor for candidemia and has been associated with poor clinical outcomes. However, in our study, the majority of patients (96.8%) had profound neutropenia at the time of candidemia diagnosis. This homogeneity limits the usefulness of ANC as a prognostic marker for mortality in this specific cohort. Although a low ALC has been associated with poor prognosis in patients with bloodstream infections, its role in predicting outcomes among pediatric patients with candidemia has not been well characterized [29,30]. In a study of 296 immunocompetent adults with candidemia, Ortega-Loubon et al. (2019) found that patients with an ALC value of <0.703 G/L at the time of candidemia diagnosis had a fivefold higher mortality risk compared to those with an ALC value of ≥0.703 G/L (odds ratio = 5.01; 95% CI, 2.39–10.93) [31]. Similarly, ALC was independently associated with 28-day mortality in our pediatric cohort. Patients with an ALC level of ≥0.2 G/L at the time of candidemia diagnosis had a significantly lower 28-day mortality rate than those with an ALC value of <0.2 G/L, with rates of 5.3% versus 50% (HR = 0.08; 95% CI, 0.01–0.61; *p* = 0.015). As ALC is a readily accessible marker routinely assessed in clinical settings, our data suggest that evaluating ALC at the time of candidemia diagnosis could aid in risk stratification and contribute to improving treatment outcomes.

In our study, patients receiving a GTX in combination with G-CSF had a lower 28-day mortality rate than those receiving G-CSF alone, with rates of 20% versus 47.4% (HR = 0.31; 95% CI, 0.11–0.85; *p* = 0.024). However, current evidence regarding the effectiveness of this treatment strategy remains inconclusive. The routine use of G-CSF remains controversial in pediatric AML patients due to a potential increased risk of relapse, which has been highlighted in several studies [32,33]. However, G-CSF could help improve neutrophil recovery, which is beneficial in life-threatening infection settings. Its use could be considered in patient with neutropenia and documented infections that are not responsive to broad-spectrum antimicrobial agents, as suggested in the expert-based recommendations of the Nordic–Dutch–Belgian–Spain–Hong Kong–Israel–Portugal consortium [34]. The RING trial (2015) reported that GTXs did not improve clinical outcomes in patients with severe infections and neutropenia (ANC < 0.5 G/L). Nevertheless, the interpretation of these results is limited by the fact that the study enrolled only half of the originally planned sample size. Moreover, post hoc analyses suggested that patients receiving GTXs at doses ≥ 0.6 G/L per transfusion may have achieved better clinical outcomes [35]. Additionally, data derived from observational studies suggest a potential clinical benefit of early GTX in neutropenic pediatric patients with life-threatening infections [36]. Therefore, based on the findings from this observational study, we hope to contribute supporting data for future analytical or prospective research.

## 5. Limitation

This study was conducted at a single institution with a relatively limited sample size.

## 6. Conclusions

Our single-center study provides epidemiological data on candidemia in one of the largest hematology centers in Vietnam. Moreover, our analysis demonstrates that an ALC ≥ 0.2 G/L, AFP, and the administration of a GTX combined with G-CSF are independent factors associated with reduced 28-day mortality in pediatric patients with acute leukemia and candidemia following intensive chemotherapy. Despite certain limitations, our findings contribute valuable clinical insights into the management of this high-risk patient population.

## Figures and Tables

**Table 1:** Baseline patient characteristics.

Characteristics		*n*	%
Sex		-	-
Male		36	57.1
Female		27	42.9
Age (years), median 9.7 (1.3–15.9)		-	-
≥10		30	47.6
<10		33	52.4
BMI		-	-
Overweight		7	11.1
Normal		45	71.4
Underweight		11	17.5
Leukemia status		-	-
R/R disease	AML	7	11.1
	ALL	21	33.3
Induction phase	AML	5	7.9
	ALL	11	17.5
Complete remission	AML	17	27
	ALL	2	3.2
Chemotherapy regimen		-	-
FLAG ± Dauno		26	41.3
FRALLE 2000 B/T Induction		10	15.9
3 + 7; ADE		10	15.9
MiDAC; HiDAC		8	12.7
COOPRALL 2007-Vanda		4	6.3
Others		5	7.9

BMI = Body mass index, R/R = Relapsed/Refractory, AML = Acute myeloid leukemia, ALL = Acute lymphoblastic leukemia.

**Table 2:** Epidemiological, laboratory, and treatment-related characteristics at the time of candidemia diagnosis.

Characteristics	*n*	%	Median (Min–Max)
*Candida* spp.	-	-	-
*C. tropicalis*	61	96.8	-
*C. albicans*	1	1.6	-
*C. krusei*	1	1.6	-
Antifungal susceptibility (*n* = 60)	-	-	-
Fluconazole	14	22.2	-
Amphotericin B	60	100	-
Caspofungin	60	100	-
Antifungal prophylaxis			-
Fluconazole	27	42.9	-
Itraconazole	11	17.5	-
No prophylaxis	25	39.7	-
Primary candidemia treatment			-
CAS	44	69.8	-
AmB	15	23.8	-
VOR + CAS	2	3.2	-
Fluconazole (Intravenous)	2	3.2	-
Granulocyte transfusions			6 units (2–12)
GTX + G-CSF	25	39.7	-
G-CSF alone	38	60.3	-
ANC at candidemia diagnosis			0.01 G/L (0–0.85)
<0.1 G/L	61	96.8	-
0.5–1 G/L	2	3.2	-
ALC at candidemia diagnosis			0.12 G/L (0–1.03)
≥0.2 G/L	19	30.2	-
<0.2 G/L	44	69.8	-
Concomitant bacterial infection			-
Yes	6	9.5	-
No	57	90.5	-

*Candida* spp. = Candida species, AmB = amphotericin B, CAS = caspofungin, VOR = voriconazole, GTX = Granulocyte transfusion, G-CSF = Granulocyte colony-stimulating factor, ANC = Absolute neutrophil count, ALC = Absolute lymphocyte count.

**Table 3:** Univariate and multivariate analyses of factors associated with 28-day mortality.

Factors	28-Day Mortality *n* (%)	Univariate Analysis		Multivariate Analysis	
		HR [95% CI]	*p*	HR [95% CI]	*p*
Fluconazole susceptibility (*n* = 60)					
Sensitive (*n* = 14)	2 (14.3)	0.3 [0.07–1.31]	0.11		
Resistant (*n* = 46)	21 (42.9)				
Antifungal prophylaxis					
Yes (*n* = 38)	10 (26.3)	0.42 [0.18–0.96]	0.04	0.31 [0.13–0.74]	0.008
No (*n* = 25)	13 (52)				
Prophylactic antifungal agents (*n* = 38)					
Fluconazole (*n* = 27)	8 (29.6)	1.85 [0.39–8.76]	0.433		
Itraconazole (*n* = 11)	2 (18.2)				
Primary treatment (*n* = 59)					
AmB (*n* = 15)	4 (26.7)	0.59 [0.2–1.76]	0.353		
CAS (*n* = 44)	18 (40.9)				
Granulocyte transfusions					
GTX + G-CSF (*n* = 25)	5 (20)	0.33 [0.12–0.9]	0.03	0.31 [0.11–0.85]	0.024
G-CSF alone (*n* = 38)	18 (47.4)				
ALC at candidemia diagnosis					
ALC ≥ 0.2 G/L (*n* = 19)	1 (5.3)	0.08 [0.01–0.6]	0.014	0.08 [0.01–0.61]	0.015
ALC < 0.2 G/L (*n* = 44)	22 (50)				

AmB = amphotericin B, CAS = caspofungin, GTX = Granulocyte transfusion, G-CSF = Granulocyte colony-stimulating factor, ALC = Absolute lymphocyte count.

## Data Availability

Data presented in this study are available upon request from the corresponding author (data are not publicly available due to privacy and ethical reasons).

## Abbreviations

The following abbreviations are used in this manuscript:

AFP	Antifungal prophylaxis
ALC	Absolute lymphocyte count
ALL	Acute lymphoblastic leukemia
AmB	Amphotericin B
AML	Acute myeloid leukemia
ANC	Absolute neutrophil count
BMI	Body mass index
CAS	Caspofungin
CI	Confidence interval
CVCs	Central venous catheters
G-CSF	Granulocyte colony-stimulating factor
GTX	Granulocyte transfusions
HR	Hazard ratio
IFI	Invasive fungal infection
R/R	Relapsed/refractory
Th17	T-helper 17
TRM	Treatment-related mortality
